# Predictive value of aorta enhancement on computed tomographic pulmonary angiography in pulmonary embolism

**DOI:** 10.1371/journal.pone.0335055

**Published:** 2025-10-24

**Authors:** Qiuyu Du, Sophie N. M. ter Haar, Jingnan Jia, Lucia J. M. Kroft, Marius Staring, Frederikus A. Klok, Berend C. Stoel

**Affiliations:** 1 Division of Image Processing, Department of Radiology, Leiden University Medical Center, Leiden, the Netherlands; 2 Department of Thrombosis and Hemostasis, Leiden University Medical Center, Leiden, the Netherlands; 3 Department of Radiology, Leiden University Medical Center, Leiden, the Netherlands; Al Nasiriyah Teaching Hospital, IRAQ

## Abstract

**Background:**

Pulmonary embolism (PE) is a life-threatening condition requiring prompt diagnosis and treatment. Visual assessment of computed tomographic pulmonary angiography (CTPA) is the first-choice diagnostic tool. New imaging biomarkers could provide additional prognostic information for improved risk stratification. We hypothesized in this exploratory study, that contrast enhancement patterns in the aorta may contain such information.

**Methods:**

CTPA scans of 93 acute PE patients were analyzed retrospectively. Firstly, the aorta was segmented automatically by TotalSegmentator and its centerline was extracted. Subsequently, lines were fitted on intensities within a region of interest perpendicularly to the aorta centerline, from which three parameters were extracted: mean intensity, proximal intensity and contrast gradient. After confounder analysis, logistic regression with forward selection evaluated the predictive value of these parameters for 12 adverse outcomes (six short-term and six long-term).

**Results:**

Lung volume, aorta dimension and contrast delay were considered as possible confounders but were not selected by forward selection. Logistic regression (n = 93) showed that a less steep contrast gradient (decreasing by 10 Hounsfield unit/%) was associated with a reduction in odds of the following short-term adverse outcomes: 48.1% for intensive care unit admission (odds ratio [OR] = 0.519, 95% confidence interval [CI]: 0.306–0.804), 29.3% for oxygen therapy >24 hours (OR = 0.707, 95% CI: 0.496–0.976), 60.6% for reperfusion therapy (OR = 0.394, 95% CI: 0.178–0.682), 57.5% for vasopressor therapy (OR = 0.425, 95% CI: 0.194–0.741), and 50.2% for PE-related death (OR = 0.498, 95% CI: 0.246–0.915). No significant associations were found with long-term outcomes.

**Conclusions:**

The aorta contrast gradient, automatically quantified from CTPA, is a relevant adjunctive predictor for short-term outcomes in PE patients. Long-term outcomes, however, could not be predicted by aorta measurement. This pilot study provides initial insights into predictive value of aorta enhancement, stimulating further exploration with external data.

## Introduction

Pulmonary embolism (PE) is a serious cardiovascular condition associated with significant morbidity and mortality [1]. PE occurs when part of a thrombus breaks off and forms an embolus in a pulmonary artery, causing a lack of blood flow and low blood oxygen levels and leading to severe complications such as shock, arrhythmia and death. These risks underscore the importance of prompt diagnosis and effective treatment to prevent poor patient outcomes.

In the diagnostic process, patients with either elevated D-dimer level and/or a high pretest probability of PE undergo computed tomographic pulmonary angiography (CTPA), which is the first-line choice for diagnosing and assessing PE due to its wide availability, rapid acquisition and ease of interpretability [2,3]. Once PE is confirmed, risk stratification—the process of classifying patients based on the severity of their PE and the likelihood of poor outcomes (such as recurrence or death)—can help clinicians to decide on the appropriate treatment. The current key prognostic determinants used for risk stratification of PE are clinical characteristics, such as hemodynamic instability, and severity of right ventricular (RV) dysfunction, commonly defined by a right ventricular-to-left ventricular diameter ratio (RV/LV) greater than 1.0. Although CTPA is primarily used to identify the presence of emboli, it can also provide valuable insights into the hemodynamic impact of the thrombus on the RV and pulmonary circulation. Investigating the correlation between CTPA characteristics and clinical outcomes could further enhance the role of CTPA as a valuable tool for initial risk stratification and targeted treatment planning.

Some studies have demonstrated that certain CT parameters of RV dimensions, pulmonary artery diameter and thrombus load hold prognostic value in PE patients and could be used in risk stratification [4,5]. It can be hypothesized that the thoracic aorta on CTPA contains additional prognostic information for PE patients, since it receives contrast media that has passed through the pulmonary circulation and both sides of the heart. Aorta enhancement on CTPA has shown its prognostic value as described in terms of the ratio between the descending aorta enhancement and the main pulmonary artery enhancement (DAE/MPAE). A study by Park et al. [6] showed that DAE/MPAE differed significantly between patients with PE-related major adverse event (MAE) and patients without MAE.

In this pilot study, we focused on contrast enhancement along the aorta because this may reflect pulmonary circulation, which influences the distribution of blood flow and the delivery of contrast media to the aorta. This enhancement could provide valuable information on restricted blood flow through the heart and lungs. Our goal was, to explore the potential of aorta enhancement on CTPA scans as a predictor of the presence of adverse clinical outcomes in PE patients. We also compared the predictive performance of contrast gradient in the aorta to two established risk stratification indicators, hemodynamic instability and RV to LV diameter ratio.

## Materials and methods

### Patients and image acquisition

This was a post-hoc analysis of a convenience cohort of 100 consecutive adult patients with CTPA confirmed acute symptomatic PE, diagnosed between July 2017 and October 2019 in the Leiden University Medical Center (LUMC), previously described [7–9]. All participant information remained anonymous throughout the study. The diagnostic workup of patients suspected of acute PE consists of a probability assessment using the PE clinical decision rules, in combination with D-dimer testing, following the YEARS algorithm [10,11]. The patients were followed for three months as part of routine clinical practice, and outcomes of care were systematically assessed and collected from patient records.

Adverse clinical outcomes included hospital or intensive care unit (ICU) admission, need for supplemental oxygen therapy or intravenous pain medication >24 hours, reperfusion therapy, vasopressor therapy, recurrent venous thromboembolism (VTE), PE-related rehospitalization and PE-related death. PE-related hospitalization was defined as readmission to the hospital due to PE-related complications, such as dyspnea, chest pain, anticoagulation-related bleeding/major bleeding or (suspected) recurrent VTE. PE-related death was defined as objectively confirmed clinically severe PE before death in the absence of an alternative diagnosis. Persistent symptoms consisted of self-reported dyspnea, chest pain and post-PE functional impairment. Post-PE functional impairment was defined as new/progressive dyspnea, exercise intolerance and/or diminished functional status following an acute PE adequately treated with anticoagulation for at least 3 months, without an apparent non-PE alternative explanation [12]. This study was approved by the institutional review board of the LUMC and informed consent requirement was waived due to its observational nature.

CTPA data was acquired on a 320-multislice detector row CT scan (Canon Aquilion ONE). Helical projection data were generated using a tube voltage of 100 peak kilovoltage (kVp) and automatic tube current modulation, 0.275-second rotation time, pitch factor of 0.813 and collimation of 80 × 0.5 mm. An iodinated contrast medium was injected-intravenously and patients were scanned during half-inspiration breath-hold. A bolus-tracking program (SureStart^TM^, Canon) was used to monitor the contrast enhancement after intravenous injection of iodinated contrast medium, to trigger scanning. The contrast injection volume and injection speed were standardized and dependent on weight category. Scanning was triggered automatically when enhancement in the pulmonary artery reached 100 Hounsfield unit (HU) above the blood pool level, with a scan delay of 5 seconds. All CT image data (512 × 512 pixels per slice) were reconstructed with Adaptive Iterative Dose Reduction 3D (AIDR3D), with 1 mm slice-thickness and 0.8 mm increment, using a lung kernel (FC08). The scanned field of view (sFOV) was 500 mm. Data were accessed for research purposes between 22/02/2024 and 03/06/2024.

### Aorta analysis

**Fig 1:**
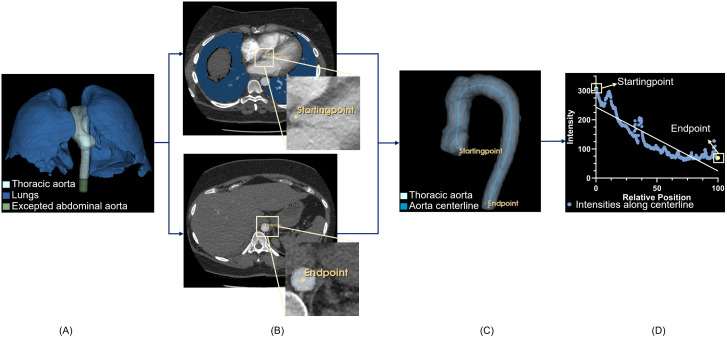
Overview of intensity measurements along of aorta centerline. A) Segmentation of the aorta and lungs via TotalSegmentator, and definition of thoracic aorta using the diaphragm as reference; B) Definition of starting- and end-points; C) Aorta centerline generated from the starting- and end-points; D) Intensity profile from the aorta centerline.

The analysis procedure contains the following steps to investigate hemodynamic changes within the thoracic aorta: 1) aorta segmentation; 2) centerline extraction, producing an intensity profile; 3) line fitting on this profile; and 4) parameter extraction from the fitted line. An overview of the intensity measurements along the aorta is shown in Fig 1.

TotalSegmentator [13,14] is an automated deep learning tool designed for comprehensive anatomical segmentation, capable of accurately delineating a wide range of anatomical structures from medical imaging modalities like CT scans. We used TotalSegmentator to segment the aorta and lungs in each CTPA scan. We extracted the thoracic section of the aorta, using the diaphragm as the reference point. Specifically, we segmented the thoracic aorta by defining the lowest plane of the extracted lung to truncate the aorta segmentation. We located the centroid of the aorta mask on this plane, referred to as “endpoint”. From this endpoint, we traced upwards along the aorta, calculating the centroid on each successive plane of the aorta mask. The lowest axial plane, in which two distinct centroids appear, was identified as the starting point of the aorta, with the ventral centroid marked as “startingpoint”. With these starting- and endpoints, we used the Vascular Modeling Toolkit in 3D Slicer to generate the aorta’s centerlines and measure its length.

To calculate the aorta intensity profile in planes perpendicularly to the local centerline, we first straightened the aorta’s centerline by curved planar reformatting in 3D Slicer, and subsequently calculated in each plane the mean intensity within the circular region of interest (ROI) in this straightened aorta.

The intensities along the aorta were further analyzed by line fitting. From these fitted lines, the following three parameters were extracted: 1) the mean intensity within the ROIs along the aorta centerline; 2) the proximal intensity, defined as the intercept of the fitted line of the aorta intensities, which is a measure of the amount of contrast at the level of the aortic root; and 3) the contrast gradient, defined as the slope of the fitted line of the aorta intensities, which is a measure of the gradient of the contrast enhancement within the aorta.

For the line fitting, the indices of the centerline coordinates served as x-axis and the corresponding CT intensities as y-axis. To compensate for the fact that the aorta length increases with age [15] and differs between patients, the indices were normalized to range from 0 to 100%.

### Statistical analysis

#### Linear regression for confounder analysis.

Analyses were conducted to identify possible confounding variables, including lung volume, aorta dimension (aorta length, aorta volume and aorta diameter) and contrast delay (the time gap between contrast injection and the start of the spiral CT scan). Specifically, lung volume and aorta volume provide insights into the influence of anatomical variations on the measurements. The aortic diameter affects flow dynamics and the volume of contrast agent which may be correlated with extracted parameters. Since an increased aortic diameter is associated with an increased risk of PE-related mortality [16], the aortic diameter was also included in our confounder analysis. Furthermore, a suboptimal contrast delay affects the quality of the enhancement of vascular structures in CT [17]. By accounting for these confounders, if needed, we aim to adjust for their effects and enable a more accurate interpretation of the primary relationships being investigated. Correlation coefficients R^2^ and *p*-values were used to evaluate the performance and significance of the regression models.

#### Firth’s penalized logistic regression for adverse outcomes predication.

Due to the low event rate in the majority of outcomes and the potential risk of small-sample bias, Firth’s penalized logistic regression [18] was conducted for each clinical outcome using forward selection based on penalized likelihood ratio (PLR) to analyze the association between our measurements and twelve clinical outcomes. With this selection method, variables that significantly improved the model were added to the model. The mean intensity, proximal intensity and contrast gradient served as independent variables. If the confounder analysis showed a significant linear correlation between independent and dependent variables, the possible confounding variables were considered covariates of the logistic regression. List-wise exclusion was used for missing values in the confounder analysis (linear regression) as well as the logistic regression. To control for multiple testing across the logistic regression models, the Benjamini–Hochberg False Discovery Rate (FDR) [19] correction was applied to the *p*-values of the selected predictors. The following results were reported per covariate for each logistic regression analysis, if applicable: beta-coefficient, odds ratio (OR), 95% confidence interval (CI) of ORs, the *p*-value of either the Wald test for variables included in the model and the *p*-value from the forward selection criterion for variables not included in the model and adjusted *p*-value by FDR correction. Furthermore, the chi-squared value of the final model and the corresponding *p*-value were reported, which tested whether the developed model differed from a model without applicable predictor variables (a model with only a constant). A *p*-value < 0.05 significance level was used to assess the results. Test statistics including sensitivity, specificity, precision and accuracy were used to evaluate the performance of predicting clinical outcomes with the extracted parameters. All statistical analyses were performed using IBM SPSS Statistics 29 and R version 4.4.1.

#### Comparison between other determinants used in risk stratification and contrast gradient.

Hemodynamic instability and RV dysfunction are two determinants in risk stratification. The mortality rate of patients presenting with high risk PE and hemodynamic instability reaches 40% within 90 days [20]. The 2019 European Society of Cardiology (ESC) guidelines [21] suggest that an elevated RV to LV diameter ratio observed on CTPA correlates with increased risk for all-cause mortality and PE-related mortality in patients with acute PE. An RV/LV greater than 1.0 is widely recognized as an indicator of poor prognosis, as it reflects RV dysfunction and has been associated with predicting short-term adverse outcomes and mortality [22,23].

In our analysis, we conducted a cross-tabulation analysis in SPSS to evaluate the associations between hemodynamic instability and adverse clinical outcomes, and between the RV/LV > 1 and adverse clinical outcomes. These analyses were used to compare the predictive performance of the contrast gradient against these two established clinical markers using evaluation metrics. To further evaluate the added prognostic value of the contrast gradient, we applied PLR tests. Specifically, we compared models that only conclude RV/LV > 1, only the contrast gradient, and both variables combined, to determine whether the contrast gradient enhances risk prediction. Lastly, we performed logistic regression analyses to investigate the correlations between hemodynamic instability and contrast gradient, and between RV/LV and contrast gradient.

## Results

### Data collection

**Fig 2:**
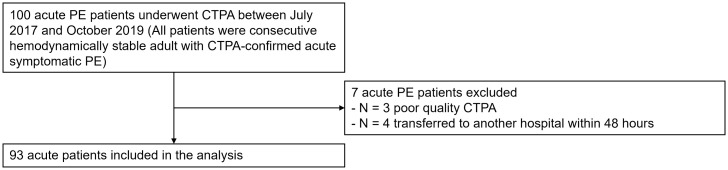
Flowchart of included patients and reasons for exclusion. PE: pulmonary embolism, CTPA: computed tomographic pulmonary angiography.

As indicated in Fig 2, three of the 100 patients were excluded due to imaging artifacts. Furthermore, four patients were transferred to another hospital within 48 hours because of logistic reasons. Therefore, both their short-term (within 7 days) and long-term outcomes were not accessible for the following analyses. The patient’s baseline characteristics is shown in Table 1.

**Table 1:** The baseline characteristics of 93 patients with acute PE.

	Total cohort
Gender (Male/Female)	50/43
Mean age (SD), years	62 (16)
Median duration of complaints (IQR), days	2 (1-7)
Recurrent VTE, N (%)	16 (17)
Provoked PE, N (%)	81 (87)
Active malignancy, N (%)	26 (28)
Immobility > 3 days or recent long travel > 6 hours in past 4 weeks, N (%)	24 (26)
Trauma/surgery during past 4 weeks, N (%)	22 (24)
Active inflammation/infection, N (%)	3 (3)
Hormone (replacement) therapy, N (%)	6 (6)
Known genetic thrombophilia, N (%)	0 (0)
Pregnancy, N (%)	0 (0)
PE Location	
Central, N (%)	33 (35)
Lobar, N (%)	9 (10)
Segmental, N (%)	41 (44)
Subsegmental, N (%)	10 (11)
Outpatient, N (%)	73 (78)
Hemodynamic instability, N (%)	5 (5)
RV/LV > 1, N (%)	44 (47)

Abbreviations: SD, standard deviation; IQR, interquartile range; VTE, venous thromboembolism; N, number of cases; PE, pulmonary embolism; RV/LV > 1: right ventricle to left ventricle diameter ratio higher than 1.

The prevalence of short-term adverse outcomes (< 7 day), long-term adverse outcomes and persistent symptoms at 3-month follow-up are presented at Table 2.

**Table 2:** The prevalence of adverse clinical outcomes and persistent symptoms in 93 patients with acute pulmonary embolism.

Total cohort (N = 93)	Positive, n (%)
**Adverse clinical outcomes < 7 days**	
Hospital admission	54 (58)
ICU admission	7 (8)
Oxygen therapy > 24 hours	23 (25)
Pain medication > 24 hours	6 (6)
Reperfusion therapy	4 (4)
Vasopressor therapy	3^#^ (3)
**Adverse clinical outcomes at 3 months**	
Recurrent VTE	1 (1)
PE-related rehospitalization	8 (9)
PE-related death	2 (2)
**Persistent symptoms at 3 months**	
Dyspnea	21 (23)
Chest pain	10 (11)
Post-PE functional impairment	21 (23)

Abbreviations: ICU, Intensive Care Unit; VTE, venous thromboembolism; PE, pulmonary embolism.

#One hemodynamically stable patient required anesthesia for intubation due to a coma unrelated to the PE and consequently received vasopressor therapy.

### Parameter extraction

**Fig 3:**
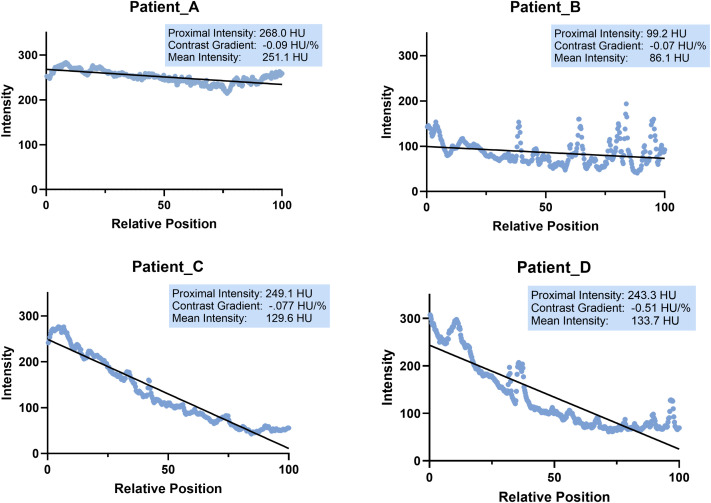
Examples of the intensity profiles along the aorta centerline, and the corresponding parameters from the line fitting. Patient A experienced dyspnea, functional impairment and chest pain during follow-up. Patient B showed no clinical adverse outcomes. Patient C required ICU admission and received thrombolysis embolectomy, oxygen therapy and vasopressor support. Patient D was also admitted to the ICU, received thrombolysis embolectomy, oxygen therapy and vasopressor support, but died subsequently due to PE related complications. Patients A and B, who showed flatter contrast gradients (i.e., less decline in intensities along aorta), had minor or no adverse outcomes while Patients C and D, who had steeper contrast gradients, experienced severe outcomes or death. The proximal intensity and mean intensity did not discriminate between mild (Patients A and B) and severe (Patients C and D) clinical outcomes.

Examples of the intensity profiles along the aorta can be found in Fig 3. On the x-axis, index 0 corresponds to the aortic root and index 100 corresponds to the end point of the thoracic aorta at the level of the diaphragm.

**Fig 4:**
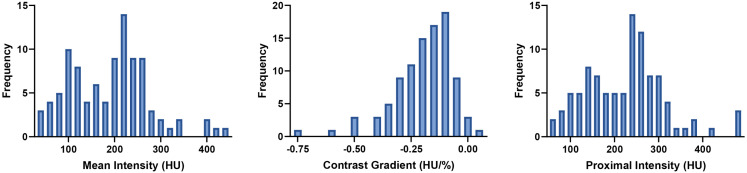
Histogram of the extracted parameters.

The histograms of the three extracted parameters are shown in Fig 4. The histogram of the contrast gradient followed a left-skewed bell-shaped distribution. The histograms of the mean intensity and the proximal amount of contrast were more similar to a bimodal distribution. No considerable outliers were observed.

### Confounder analysis

The results of the confounder analysis are shown in Table 3. As there were no missing values in the independent or dependent variables, all 93 patients were included in this analysis. All independent variables showed significant correlations (*p*-value of ANOVA testing was smaller than 0.05) with mean intensity and proximal intensity, while only three of the independent variables (aorta length, aorta volume, and aorta diameter) showed significant correlations with contrast gradient. Although the correlations were weak, since the R^2^-values were relatively low (ranging from 0.052 to 0.222), the dependent variables were considered potential confounders and were included as covariates in the logistic regression analysis. As the β-coefficients were negative (except for the contrast delay), a higher value of independent variables corresponded to a lower value of dependent variables.

**Table 3:** Results of the confounder analysis of the dependent variable, including lung volume, length of aorta, volume of aorta, diameter of aorta and contrast delay.

	Mean Intensity				Proximal Intensity				Contrast Gradient			
	R²	*p*	β	95% CI	R²	*p*	β	95% CI	R²	*p*	β	95% CI
Lung Volume	0.156	<.001*	−0.026	[−.038, −.014]	0.106	.001*	−0.022	[−.035, −.009]	0.013	0.259	−1.2E-5	[−3.2E-5, 8.6E-6]
Aorta Length	0.121	<.001*	−0.676	[−1.047, −.035]	0.052	.024*	−0.457	[−.853, .061]	0.064	0.012*	−7.5E-4	[−1.0E-3, −1.6E-4]
Aorta Volume	0.222	<.001*	−5.3E-4	[−7.3E-4, −3.3E-4]	0.129	<.001*	−4.1E-4	[−6.3E-4, −1.9E-4]	0.063	0.013*	−4.3E-7	[−7.6E-7, −9.2E-8]
Aorta Diameter	0.192	<.001*	−11.098	[−15.730, −6.466]	0.116	<.001*	−8.859	[−13.843, −3.875]	0.062	0.014*	−0.010	[−.017, −.002]
Contrast Delay	0.074	0.007*	0.329	[0.092, 0.566]	0.072	.008*	0.333	[0.089, 0.577]	0.000	0.901	−2.3E-5	[−4.0E-4, 3.5E-4]

Abbreviations: R^2^, R square; *p*, *p*-value for the significance of the independent variable; β, beta-coefficient; CI, confidence interval.

### Logistic regression analysis

Table 4 presents the result of the Firth’s logistic regression for the clinical outcomes, where contrast gradient was the only predictive variable selected by the forward selection, emerged as a significant predictor of five out of twelve clinical outcomes. To improve interpretability, the contrast gradient was rescaled by a factor of 10, so that the reported odds ratios correspond to a 10-unit decrease (HU/%) in contrast gradient. Under this scaling, a less steep contrast gradient was significantly associated with a 48.1% decrease in the odds of admission to ICU (OR = 0.519, 95% CI: 0.306–0.804), a 29.3% decrease in the odds of oxygen therapy > 24 hours requirements (OR = 0.707, 95% CI: 0.496–0.976), a 60.6% decrease in the odds of reperfusion therapy (OR = 0.394, 95% CI: 0.178–0.682), a 57.5% decrease in the odds of vasopressor therapy (OR = 0.425, 95% CI: 0.194–0.741) and a 50.2% decrease in the odds of PE-related death (OR = 0.498, 95% CI: 0.246–0.915). Scatter plots of these five outcomes against the contrast gradient can be found in S1 Fig, in the supplement. Chi-square tests were conducted to determine, whether the models developed with the predictive variables differed significantly from the base model without those variables. The Chi-square tests shown in Table 4 indicate that the predictors included in the models had a considerable impact on the outcomes (p < 0.05). As reported in Table 4, the FDR-adjusted *p*-values of contrast gradient showed statistical significance.

**Table 4:** Results of the logistic regression for the short-term outcomes, for which a predictive variable was selected in the forward selection.

	ICU admission (n = 93)				Oxygen therapy (n = 93)				Reperfusion therapy (n = 93)				Vasopressor (n = 93)				PE-related death (n = 90)			
	β	OR (95% CI)	p	p-adjusted	β	OR (95% CI)	p	p-adjusted	β	OR (95% CI)	p	p-adjusted	β	OR (95% CI)	p	p-adjusted	β	OR (95% CI)	p	p-adjusted
Constant	−4.082	0.017 (0.002, 0.070)	<.001*	NA	−1.826	0.161 (0.062, 0.371)	<.001*	NA	−5.659	0.003 (0.000, 0.028)	<.001*	NA	−5.704	0.003 (0.000, 0.029)	<.001*	NA	−5.450	0.004 (0.000, 0.039)	<.001*	NA
Contrast gradient	−0.656	0.519 (0.306, 0.804)	0.004*	0.006*	−0.347	0.707 (0.496, 0.976)	0.035*	0.035*	−0.931	0.394 (0.178, 0.682)	<.001*	0.003*	−0.855	0.425 (0.194, 0.741)	0.003*	0.006*	−0.698	0.498 (0.246, 0.915)	0.028*	0.035*
Chisq of model (Sig.)	11.043(<.001*)				7.915(0.005*)				13.643(<.001*)				10.743(0.001*)				6.412(0.011*)			

Abbreviations: ICU, Intensive Care Unit; PE, pulmonary embolism; β, beta-coefficients; p, p-value for the significance of the predictor; Chisq, Chi-square; Sig., significance of the logistic regression model; p-adjusted: p-values were adjusted using the Benjamini–Hochberg false discovery rate (FDR) method; OR: odds ratio; CI: confidence interval.

*: *p* < 0.05. Please note that the *p*-values of the parameters included in the model are based on the Wald test, whereas the *p*-values of parameters excluded from the model result from the forward selection criterion.

Additionally, all β-coefficients for the contrast gradient in Table 4 were negative. This suggests that a steeper decline in intensity along the aorta was associated with a higher likelihood of ICU admission, extended oxygen therapy, reperfusion therapy, vasopressor therapy and PE-related death.

S1 Table contains the results for the outcomes, for which no predictive variables were selected by forward selection method. The PLR forward selection found no predictive variable in seven out of twelve analyses, meaning that the extracted parameters were not significantly associated with the following seven outcomes: hospital admission, pain medication > 24 hours, PE-related rehospitalization, post-PE dyspnea, post-PE chest pain, recurrent VTE and post-PE functional impairment.

### Comparison of contrast gradient with RV to LV ratio and hemodynamic instability

We compared the performance of predicting adverse clinical outcomes between contrast gradient, hemodynamic instability and RV/LV > 1, which are well-established biomarkers for predicting these outcomes in PE patients. Table 5 presents the result of evaluation metrics (sensitivity, specificity, positive predictive value and accuracy) for the contrast gradient, hemodynamic instability and RV/LV > 1 in predicting adverse clinical outcomes. S2 Fig shows the receiver operating characteristic (ROC) curves for contrast gradient when predicting the presence of short-term clinical outcomes.

**Table 5:** Comparison results between contrast gradient, hemodynamic instability and RV/LV > 1 when predicting the presence of adverse clinical outcomes.

	Contrast Gradient					Hemodynamic instability					RV/LV > 1				
	SEN	SPC	PPV	ACC(%, ^a^)	*p*	SEN	SPC	PPV	ACC (%)	*p*	SEN	SPC	PPV	ACC(%)	*p*
ICU admission	14.3	100	100	93.5	0.004*	100	97.7	71.4	97.8	<.001*	14.3	98.8	50.0	92.5	<.001*
Oxygen Therapy	8.7	97.1	50.0	75.3	0.034*	100	79.5	21.7	80.6	<.001*	8.7	95.5	33.3	73.4	0.004*
Reperfusion therapy	25.0	100	100	96.8	<.001*	80.0	100	100	98.9	<.001*	25.0	100	100	96.8	<.001*
Vasopressor therapy	33.3	100	100	97.8	0.003*	40.0	98.9	66.7	95.7	<.001*	33.3	100	100	97.8	0.007*
PE-related death	0	100	100	97.8	0.036*	40.0	100	100	96.7	<.001*	**–**	**–**	**–**	**–**	**–**

Abbreviations: RV/LV > 1, right ventricle to left ventricle diameter ratio higher than 1; SEN, sensitivity; SPC, specificity; PPV, positive predictive value; ACC, accuracy; *p*, *p*-value for the significance of the predictor; ICU, Intensive Care Unit; PE, pulmonary embolism.

^a^: the cut value is 0.5.

Table 5 illustrates that RV/LV > 1 performed comparably to the contrast gradient in predicting reperfusion therapy and vasopressor therapy. However, for ICU admission and oxygen therapy, RV/LV > 1 exhibited a slight disadvantage, as indicated by lower specificity and positive predictive value (PPV) compared to the contrast gradient. Additionally, no significant correlation was observed between an RV/LV > 1 and PE-related death. Detailed results of the PLR tests, including likelihoods, Chi-square values, and p-values, which compare models incorporating the contrast gradient and the RV/LV > 1 in predicting adverse outcomes, are presented in S3 Table.

As shown in Table 5, hemodynamic instability is a stronger predictor than contrast gradient of most adverse clinical outcomes, except for predicting vasopressor therapy. S2 Table presents the results of cross-tabulation analyses for hemodynamic instability and long-term adverse outcomes, showing no significant correlation. Additionally, the correlation between hemodynamic instability and the contrast gradient, and between RV/LV > 1 and the contrast gradient are illustrated in S4 Table.

## Discussion

### Study findings

In this study, we developed a standardized and deterministic image processing method, to automatically measure contrast enhancement patterns in the aorta from CTPAs. In a group of 93 patients diagnosed with acute PE, we found that the contrast gradient in the aorta correlated with short-term adverse clinical outcomes and PE-related death. In logistic regression analyses, a negative β-coefficient in logistic regression analyses between contrast gradient and adverse clinical outcomes indicated that a steeper drop in intensities along aorta centerline was associated with a higher probability of admission to ICU, need for oxygen therapy > 24h, reperfusion therapy, vasopressor therapy and suffering from PE-related death.

Although the contrast gradient demonstrated higher specificity and PPV as compared to RV/LV across short-term outcomes, its sensitivity was limited. Therefore, contrast gradient is not a stand-alone predictor, but should be positioned as an adjunctive imaging marker in combination with established predictors such as hemodynamic instability.

In general, patients with a preexisting heart conditions or higher thrombus load are at greater risk for these adverse clinical outcomes [21,24]. Since contrast is administered as a bolus, and bolus progression will be slower in patients with compromised circulation, the bolus may not have reached the distal aorta as much as in patients without compromised circulation. This can cause a larger difference between the enhancement in the proximal ascending aorta as compared to the distal aorta, and hence a steeper slope, while in patients without compromised circulation the bolus has progressed fully into the aorta, resulting in a flatter slope. The same effect could come from decreased circulation through the lungs caused by a higher thrombus load, also resulting in a steeper slope. These hypotheses on hemodynamic interpretations need to be confirmed in future studies including physiological modeling of the combined effects of decreased lung circulation and decreased cardiac output.

### RV to LV ratio

Results shown in Table 5 suggest that, compared with RV/LV, the contrast gradient demonstrated higher specificity and PPV across short-term clinical outcomes, indicating its stronger ability to specifically identify patients with adverse outcomes. S3 Table shows that for five clinical outcomes, the full model including both contrast gradient and RV/LV > 1 significantly outperformed models that excluded any variable (all *p* < 0.001). Specifically, the removal of the contrast gradient resulted in larger reduction in model fit than removing RV/LV > 1, suggesting that the contrast gradient captures additional prognostic information not fully explained by RV dysfunction alone. Furthermore, as shown in S4 Table, we observed a negative correlation between RV/LV > 1 and the contrast gradient, with a negative β-coefficient indicating that a steeper decline in the contrast gradient was associated with a higher likelihood of RV/LV > 1. This suggests that the contrast gradient may reflect the hemodynamic impact of PE on the cardiovascular system, and may serve as additional marker for compromised circulation as risk factor for complications. However, similar to the contrast gradient, RV/LV did not show a significant correlation with long-term adverse clinical outcomes.

### Hemodynamic instability

Blood pressure levels fluctuate frequently, resulting in numerous records for a patient upon arrival at the emergency department. Clinicians typically select one of these records to assess the patient’s blood pressure status. Patients with extremely low blood pressure are too unstable to undergo a CT scan and will therefore often be diagnosed with an echocardiogram showing an enlarged right ventricle. Therefore, the hemodynamically most unstable patients were not included in this study. Hemodynamic instability correlated with a higher likelihood of the presence of adverse short-term outcomes, as illustrated in Table 5. Hemodynamic instability is the indication for vasopressor therapy. However, as shown in Table 5, its predictive performance for vasopressor therapy was inferior to that of the contrast gradient. This discrepancy may be due to variations in clinical decision-making, as not all hemodynamically unstable patients received vasopressor therapy. Furthermore, one hemodynamically stable patient in our database required vasopressors due to intubation. Similar to the contrast gradient, hemodynamic instability does not show a correlation with long-term adverse outcomes. However, it is correlated with hospital admissions, albeit with relatively low sensitivity and accuracy, as shown in S2 Table. Additionally, the correlation between hemodynamic instability and the contrast gradient, illustrated in S4 Table, suggests that the contrast gradient derived from CT images automatically has the potential to serve as a predictor for poor outcomes in patients with acute PE.

### Limitations and future work

All CTPAs included in our dataset were acquired at a single center using a 320-detector row CT scanner with standardized scan acquisition and contrast administration protocols. While this ensured internal consistency, it may limit the external applicability of the findings. Results may vary across different scanner types, manufacturers, acquisition protocols, and institutional practices. These technical factors can influence the intensity values within the aorta, potentially affecting the robustness and generalizability of the statistical outcomes. Future studies should therefore include multicenter data from different scanners and protocols to validate and generalize the findings.

Moreover, the sample size was limited, with only 93 patients included in this study. Some outcome measures were reported for only a few patients: recurrent VTE (1 patient), PE-related death (2 patients), vasopressor therapy (3 patients), reperfusion therapy (4 patients), pain medication >24 hours (6 patients), ICU admission (7 patients) and PE-related rehospitalization (8 patients). This limited sample size may have contributed to the absence of significant differences, especially if the effect size was limited.

Additionally, as shown in Fig 3, the measurements contain a periodical fluctuation. This could be due to dense contrast artifacts in the superior vena cava and the spine around lower part of aorta. The presence of calcium within the aorta also results in a higher intensity compared to normal regions. Future study could focus on developing a method to correct for this fluctuation, improving the accuracy of the measurement.

This was a proof-of-concept study. Larger, external cohorts consisting of hemodynamically stable patients with PE will be essential to validate the prognostic significance of aortic enhancement in the patient group where these measures can help risk stratification. For a patient presenting in shock, this is less relevant as the indication for ICU admission and reperfusion treatment is clear. While our analysis demonstrates that the contrast gradient adds significant prognostic value alongside the RV/LV, its prognostic capacity could be further evaluated by comparisons with widely adopted clinical risk models such as the Pulmonary Embolism Severity Index (PESI) or simplified PESI (sPESI). Additionally, this study only considered the enhancement intensities within the aorta, while intensities in other vascular structures or tissues may also provide relevant insights. Furthermore, it is possible that there are confounders at play that were unaccounted in this study, such as the volume and injection speed of the contrast agent administration. Future research should investigate integrative models that combine contrast gradient with other CT-based parameters or clinical variables to further enhance predictive modeling. Given that the contrast gradient has shown predictive value across multiple outcomes, this parameter should be investigated in subsequent studies and potentially incorporated into predictive models for PE patient outcomes.

## Conclusion

This proof-of-concept study indicates that the contrast gradient along the aorta may be a relevant predictor of short-term adverse clinical outcomes in patients with acute PE. Although our findings should be regarded as hypothesis generating, it has the potential to serve as an automatically obtainable and consistent parameter for risk stratification and treatment decision-making, thereby expanding the utility of CTPA in the management of acute PE and related conditions.

## Supporting information

S1 Fig Scatter plots of the contrast gradient versus the outcomes for which the contrast gradient is a significant predictor. ICU: Intensive Care Unit, PE: Pulmonary Embolism, VTE: Venous Thromboembolism.
(TIF)

S2 Fig The ROC curve of the contrast gradient, hemodynamic instability and RV/LV > 1 when predicting the presence of short-term clinical outcomes. SEN: sensitivity; SPC: specificity.
(TIF)

S1 Table Results of the logistic regression for outcomes for which no predictive variable was selected in the forward selection. n, Number of Patients Included for Analysis; PE, Pulmonary Embolism; VTE, venous thromboembolism; p, p-value; CI, confidence interval. Please note that the p-values of parameters excluded from the model result from the forward selection criterion.
(DOCX)

S2 Table Results of cross-tabulation analyses for hemodynamic instability and adverse outcomes with no significance, except for hospital admission. SEN, sensitivity; SPC, specificity; PPV, positive predictive value; ACC, accuracy; *p*: *p*-value; PE, pulmonary embolism; VTE, venous thromboembolism.
(DOCX)

S3 Table Penalized likelihood ratio test comparing contrast gradient and RV/LV ratio in predicting adverse clinical outcomes. PE, pulmonary embolism; ICU, Intensive Care Unit; RV/LV > 1, right ventricle to left ventricle diameter ratio higher than 1; Chisq, Chi-square; NA: not applicable; w/o, without.
(DOCX)

S4 Table Result of the logistic regression for hemodynamic instability and the contrast gradient, and for RV/LV and contrast gradient. RV/LV > 1, right ventricle to left ventricle diameter ratio higher than 1; β, beta-coefficient;
(DOCX)
