# Supplementary figures and images for: Predictive value of aorta enhancement on computed tomographic pulmonary angiography in pulmonary embolism
Source: PLoS One. 2025 Oct 24;20(10):e0335055. doi: 10.1371/journal.pone.0335055 (PMC12551865; doi:10.1371/journal.pone.0335055)

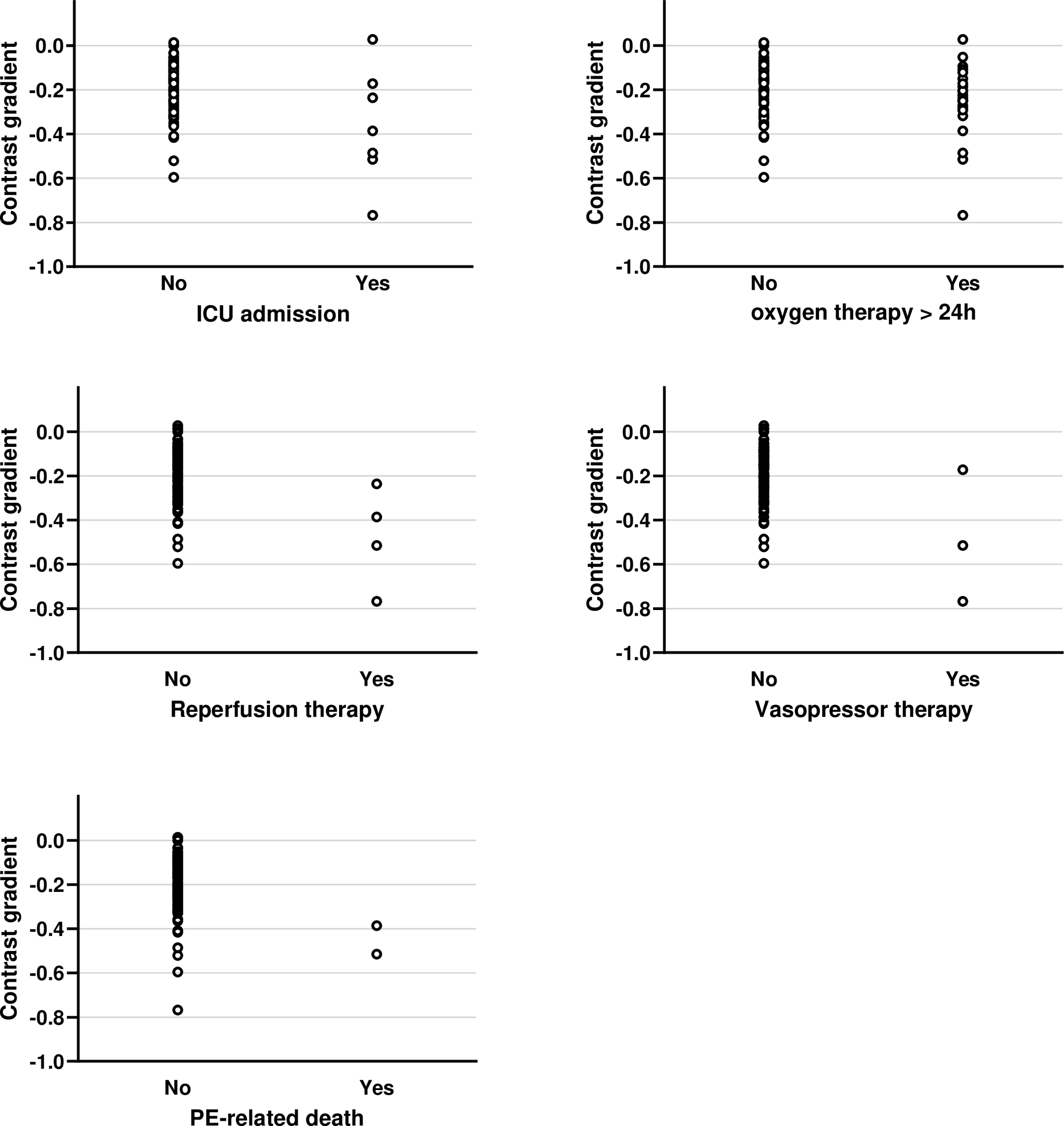

Supplement: S1 Fig — ICU: Intensive Care Unit, PE: Pulmonary Embolism, VTE: Venous Thromboembolism. (TIF) [file pone.0335055.s001.tif]

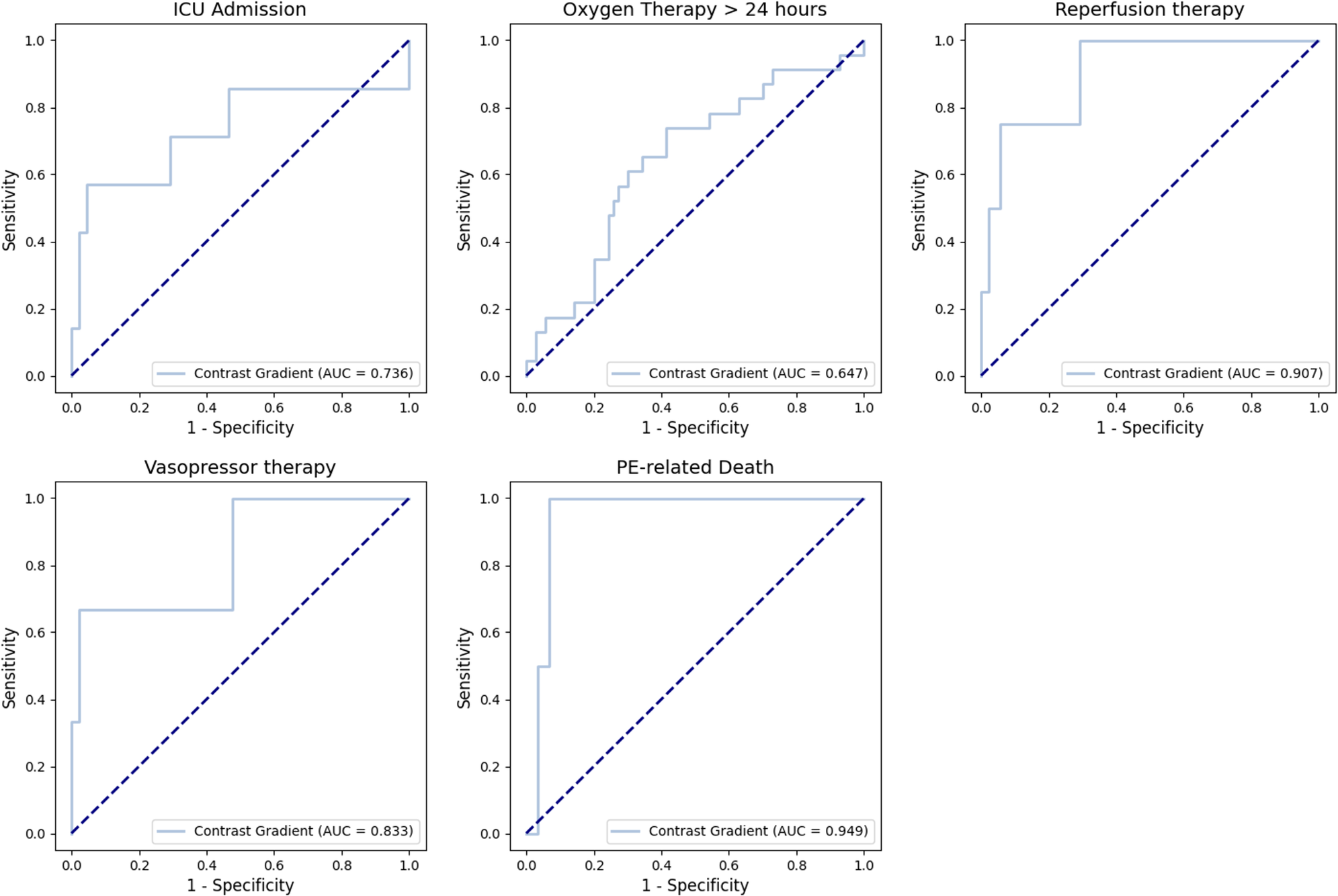

Supplement: S2 Fig — SEN: sensitivity; SPC: specificity. (TIF) [file pone.0335055.s002.tif]
