# Supplementary material for: Predictive value of aorta enhancement on computed tomographic pulmonary angiography in pulmonary embolism
Source: PLoS One. 2025 Oct 24;20(10):e0335055. doi: 10.1371/journal.pone.0335055 (PMC12551865; doi:10.1371/journal.pone.0335055)
Supplement: S1 Table — n, Number of Patients Included for Analysis; PE, Pulmonary Embolism; VTE, venous thromboembolism; p, p-value; CI, confidence interval. Please note that the p-values of parameters excluded from the model result from the forward selection criterion. (DOCX) [file pone.0335055.s003.docx]

|  | Short-term | | Long-term adverse clinical outcomes | | | | |
| --- | --- | --- | --- | --- | --- | --- | --- |
|  | Hospital admission  (n = 93) | Pain medication  > 24h  (n = 93) | Dyspnea  (n = 90) | Post-PE functional impairment  (n = 90) | Post-PE chest pain  (n = 90) | Recurrent VTE  (n = 90) | PE-related rehospitalization  (n = 88) |
|  | *p* | *p* | *p* | *p* | *p* | *p* | *p* |
| Mean intensity | 0.324 | 0.070 | 0.943 | 0.808 | 0.135 | 0.281 | 0.547 |
| Proximal intensity | 0.167 | 0.192 | 0.960 | 0.720 | 0.194 | 0.432 | 0.788 |
| Contrast gradient | 0.078 | 0.126 | 0.799 | 0.780 | 0.788 | 0.389 | 0.365 |
| Lung volume | 0.263 | 0.253 | 0.668 | 0.566 | 0.759 | 0.958 | 0.410 |
| Log of lung volume | 0.203 | 0.274 | 0.893 | 0.732 | 0.598 | 0.905 | 0.793 |
| Aorta length | 0.209 | 0.095 | 0.592 | 0.505 | 0.647 | 0.734 | 0.184 |
| Aorta volume | 0.611 | 0.535 | 0.148 | 0.431 | 0.687 | 0.132 | 0.179 |
| Log of aorta volume | 0.714 | 0.277 | 0.185 | 0.436 | 0.004* | 0.172 | 0.158 |
| Aorta diameter | 0.900 | 0.711 | 0.098 | 0.454 | 0.569 | 0.069 | 0.208 |
| Contrast delay | 0.299 | 0.778 | 0.494 | 0.501 | 0.717 | 0.863 | 0.678 |

* p < 0.05. The log-transformed aorta volume showed a significant correlation with post-PE chest pain, which may be due to chance and lacks a clear clinical explanation.
