# Supplementary material for: Predictive value of aorta enhancement on computed tomographic pulmonary angiography in pulmonary embolism
Source: PLoS One. 2025 Oct 24;20(10):e0335055. doi: 10.1371/journal.pone.0335055 (PMC12551865; doi:10.1371/journal.pone.0335055)
Supplement: S2 Table — SEN, sensitivity; SPC, specificity; PPV, positive predictive value; ACC, accuracy; p: p-value; PE, pulmonary embolism; VTE, venous thromboembolism. (DOCX) [file pone.0335055.s004.docx]

|  | Hemodynamic instability | | | | |
| --- | --- | --- | --- | --- | --- |
|  | SEN | SPC | PPV | ACC(%) | *p* |
| Post-PE Chest Pain (n = 90) | 10.0 | 95.0 | 20.0 | 85.6 | 0.515 |
| Dyspnea (n = 90) | 9.5 | 95.7 | 40.0 | 75.6 | 0.365 |
| Recurrent VTE (n = 90) | 0 | 84.4 | 0 | 93.3 | 0.807 |
| PE-related rehospitalization (n = 88) | 0 | 93.8 | 0 | 85.2 | 0.467 |
| Post-PE functional impairment (n = 90) | 9.5 | 95.7 | 40.0 | 75.6 | 0.365 |
| Pain medication > 24h (n = 93) | 0 | 94.3 | 0 | 88.2 | 0.546 |
| Hospital admission (n = 93) | 10.3 | 100 | 100 | 46.4 | 0.038* |

*: *p* <0.05.
