# Supplementary material for: Predictive value of aorta enhancement on computed tomographic pulmonary angiography in pulmonary embolism
Source: PLoS One. 2025 Oct 24;20(10):e0335055. doi: 10.1371/journal.pone.0335055 (PMC12551865; doi:10.1371/journal.pone.0335055)
Supplement: S3 Table — PE, pulmonary embolism; ICU, Intensive Care Unit; RV/LV > 1, right ventricle to left ventricle diameter ratio higher than 1; Chisq, Chi-square; NA: not applicable; w/o, without. (DOCX) [file pone.0335055.s005.docx]

| Model comparison | PE-related death | | | ICU admission | | | Oxygen therapy | | | Reperfusion therapy | | | Vasopressor | | |
| --- | --- | --- | --- | --- | --- | --- | --- | --- | --- | --- | --- | --- | --- | --- | --- |
|  | Model LogLik | Chisq | *p* | Model LogLik | Chisq | *p* | Model LogLik | Chisq | *p* | Model LogLik | Chisq | *p* | Model LogLik | Chisq | *p* |
| Full model | -6.12 | NA | NA | -17.35 | NA | NA | -43.05 | NA | NA | -8.29 | NA | NA | -7.51 | NA | NA |
| w/o contrast gradient | -36.98 | 61.72 | <.001* | -47.77 | 60.83 | <.001* | -59.08 | 32.06 | <.001* | -43.88 | 71.18 | <.001* | -38.99 | 62.95 | <.001* |
| w/o RV/LV >1 | -23.42 | 34.60 | <.001* | -40.69 | 46.69 | <.001* | -55.85 | 25.60 | <.001* | -35.57 | 54.56 | <.001* | -31.13 | 47.23 | <.001* |

Abbreviation: PE, pulmonary embolism; ICU, Intensive Care Unit; RV/LV > 1.0, right ventricle to left ventricle diameter ratio higher than 1; Chisq, Chi-square; NA: not applicable; w/o, without.

Full models include both covariates (the contrast gradient and RV/LV > 1.0).

w/o variates model fix the mentioned variate to zero.
