# Supplementary material for: Predictive value of aorta enhancement on computed tomographic pulmonary angiography in pulmonary embolism
Source: PLoS One. 2025 Oct 24;20(10):e0335055. doi: 10.1371/journal.pone.0335055 (PMC12551865; doi:10.1371/journal.pone.0335055)
Supplement: S4 Table — RV/LV > 1, right ventricle to left ventricle diameter ratio higher than 1; β, beta-coefficient; (DOCX) [file pone.0335055.s006.docx]

|  | Hemodynamically instable  (n = 93) | | RV/LV >1.0  (n = 93) | |
| --- | --- | --- | --- | --- |
|  | β | *p* | β | *p* |
| Constant | -5.706 | <.001* | -1.254 | 0.004* |
| **Contrast Gradient^#^** | **-1.048** | **0.002*** | **-0.613** | **0.002*** |
| Chi-square of model (Sig.) | 14.380(<.001*) | | 12.175(<0.001*) | |

*: p<0.05

^#^: The contrast gradient was multiplied by 10 to match the unit scale used in the main text.
